# Revisiting Link Quality Metrics and Models for Multichannel Low-Power Lossy Networks

**DOI:** 10.3390/s23031303

**Published:** 2023-01-23

**Authors:** Jing Mao, Yan Zhao, Yu Xia, Zhuopeng Yang, Cheng Xu, Wei Liu, Daqing Huang

**Affiliations:** 1 School of Electrical and Electronic Engineering, Chongqing University of Technology, Chongqing 400054, China; 2 China Aerospace Academy of Systems Science and Engineering, Beijing 100037, China; 3 College of Electronic and Information Engineering, Nanjing University of Aeronautics and Astronautics, Nanjing 210016, China

**Keywords:** link quality, multichannel, single channel, interference, low-power lossy network, packet reception ratio (PRR), physical layer metrics

## Abstract

Multichannel communication has great potential in environments with unknown interference patterns. However, existing link quality metrics and models are generally established and verified under a single-channel scenario, which does not consider the impacts of radio interference and channel change. Therefore, it is hard to directly judge whether these metrics and models are still valid under a multichannel scenario. This paper empirically analyzes the applicability of popular link quality metrics and models in multiple channels with different interference levels. Results show that the link quality estimation (LQE) capability of traditional metrics will be affected by the interference level of the channel, which makes the conclusions obtained under a single-channel scenario no longer valid. Meanwhile, traditional LQE models are basically not adaptive to radio interference and channel change. They are only valid for channels with similar interference under which they are modeled. If these models are directly used under a multichannel scenario, the link quality will be overestimated inevitably. In other words, traditional LQE metrics and models cannot be directly used in the multichannel scenario. It is necessary to deeply analyze the statistical characteristics of popular link quality metrics in multiple typical channels and design channel and interference adaptive metrics and models to support effective multichannel communication.

## 1. Introduction

Low-power Lossy Network (LLN), such as Wireless Sensor Network (WSN), comprised of many low-cost and low-power nodes aims to support various applications, such as military surveillance, environment monitoring, and factory automation [1]. At its early stage, it commonly uses single-channel protocols for simple operation on resource-constrained nodes [2]. However, as the Industrial Scientific Medical (ISM) frequency band in which LLN generally stays is also occupied by many other wireless technologies (e.g., WiFi and Bluetooth), the performance of single-channel communication will be impaired inevitably. To improve the reliability of LLN, multichannel communication, especially the Time Slotted Channel Hopping (TSCH) technology, has received extensive attention [3,4,5,6,7,8], which has the potential to be a link-layer solution for LLN due to its resilience to wireless interference and multi-path fading.

The multihop and self-organization characteristics of LLN determine that its topology and link quality have great impacts on the network performance. To combat the inherent dynamic changes of low-power wireless links, agile and accurate link quality estimation (LQE) is essential. There have been many works in the literature which analyze the characteristics of low-power wireless links through experiments and propose many effective LQE metrics (e.g., Packet Reception Ratio (PRR), Signal to Noise Ratio (SNR), Received Signal Strength Indicator (RSSI) and Link Quality Indicator (LQI)) and models [9]. However, almost all these metrics and models are established and verified under the single-channel scenario [10,11,12,13,14,15,16,17,18,19,20], and most of them are under the interference-free channel [10,11,12,13,14,15,16,18,19]. The impacts of radio interference and channel change are not fully considered. Therefore, it is hard to directly judge whether these metrics and models are still valid under a multichannel scenario.

In this paper, the applicability of popular LQE metrics and models in many different channels is analyzed empirically. Results show that the interference level of the channel will affect the LQE capability of traditional metrics. LQI, which is thought to have the highest resolution in traditional LLN, is inferior to SNR and RSSI under interfered channels. In particular, its LQE capability has even been completely lost in channels with strong interference. On the other hand, traditional LQE models including theoretical and empirical models are basically not adaptive to radio interference and channel change. They are only valid for channels with similar interference under which they are modeled. Therefore, traditional LQE metrics and models cannot be directly used in the multichannel scenario. On the one hand, the thresholds of popular link quality metrics established in traditional LLN will lead to incorrect judgment of link quality under a multichannel scenario. On the other hand, the link quality will be overestimated inevitably if traditional link quality models are directly used under a multichannel scenario. It is necessary to study and design channel and interference adaptive LQE metrics and models.

The main contributions are as follows: (1) The applicability of popular LQE metrics and models in multiple different channels are analyzed empirically. (2) The problems of these metrics and models directly used under multichannel scenarios are pointed out. (3) The conclusions could provide new insights for multichannel protocol design. To the best knowledge of the authors, this is the first time to discuss the applicability of traditional link quality metrics and models under a multichannel scenario.

The rest of this paper is structured as follows. In Section 2, the works related to LQE and multichannel communication are described. Popular link quality metrics and models are summarized and their mathematical descriptions are presented in Section 3. This is followed by the experimental setup in Section 4, including the experimental field, channel selection, and test methodology. Section 5 analyzes the spatial characteristics of low-power wireless links under different channels and evaluates the applicability of main spatial models. Section 6 analyzes the performance of popular physical layer metrics and models under different channels. Section 7 evaluates the generality of the conclusions acquired in Section 6 with a completely different experimental setup. Finally, conclusions are presented and suggestions are made for future works.

## 2. Related Works

### 2.1. Link Quality Estimation for Single Channel

For traditional LLN, link characteristic analysis and link quality estimation have fundamental impacts on the network performance and also affect the design of higher-layer protocols. Therefore, they have attracted a lot of research efforts in the past decade. Existing works have shown that the quality of low-power wireless links changes timely and spatially. Meanwhile, there is an asymmetry between uplinks and downlinks [9,18]. Moreover, node mobility or cross-technology interference (CTI) will also affect the accuracy of LQE. Wen et al. [19] showed that low-power links are highly sensitive to node mobility and predicting the existence and range of transitional regions is more difficult. Barać et al. [20] showed that LQI and RSSI obtained from interfered WSN links are deceptive and cannot be used to assess link quality accurately.

As low-power wireless links are unstable, accurate LQE is necessary for efficient routing and transmission [9]. PRR is the most direct metric for LQE. However, as it always takes a long time to get accurate PRRs, the agility of using PRR directly is very poor [9]. To describe the spatial variation of PRR, its spatial distribution model with distance could be established based on the lognormal path loss model [10]. However, Liu et al. confirmed that this model is very inaccurate [21]. Despite all this, the bounds of the transitional region could be estimated using the spatial distribution model of PRR with distance [22], which is valuable for network planning and node deployment.

Physical layer metrics such as SNR, RSSI, and LQI are also widely used in LQE, due to their high correlation with PRR [9]. Sun et al. proposed to estimate the link quality using the theoretical model of SNR and PRR [11]. Liu et al. established a fitting model of SNR and PRR using logical regression [12]. Also using logistic regression, Ye et al. established a fitting model of RSSI and PRR [13]. Liu et al. proposed a simplified theoretical model of RSSI and PRR for self-adaptive LQE, which greatly reduces the computational overhead of the traditional theoretical model [14]. Gomes et al. established a polynomial mapping model between the normalized RSSI and PRR for LQE [17]. Carles et al. and Luo et al. established the multi-segment linear model and cubic fitting model of LQI and PRR respectively [15,16]. Liu et al. also established a model of LQI and PRR using logical regression and pointed out that it is more accurate than those of SNR, RSSI, and PRR [12]. Almost all the works above are established and verified under a single-channel scenario [10,11,12,13,14,15,16,17,18,19,20], and most of them are under the interference-free channel [10,11,12,13,14,15,16,18,19]. The impacts of radio interference and channel change are not fully considered.

### 2.2. Multichannel Communication

To improve the reliability of LLN, multichannel communication has received extensive attention in recent years [3,4,5,6,7,8]. Wu et al. utilized multiple channels to improve communication performance and proposed a tree-based, multichannel scheme for data collection applications, which allocates channels to disjoint trees and exploits parallel transmissions among trees. Experimental results showed that the proposed scheme significantly improves the network throughput and reduces packet losses [3]. Tang et al. proposed an adaptive receiver-initiated multichannel rendezvous mechanism, in which every node dynamically optimizes the selection of wireless channels based on the channel conditions it senses. Experimental results showed that the proposed mechanism substantially enhances wireless channel utilization and transmission efficiency while resisting wireless interference and jamming [4]. Zanella et al. improved the accuracy of received signal strength-based ranging by averaging the received signal strength samples collected on different channels, as multichannel averaging can reduce the received signal strength variability caused by multipath propagation [5].

By using receiver-side load estimation without any explicit control message exchange, Kim et al. proposed an autonomous and adaptive TSCH slot allocation scheme that adjusts the number of slots per frame according to varying traffic loads, which significantly enhances the performance of autonomous scheduling algorithms [6]. Aiming at bringing TSCH to low-power iPv6 networks, Duquennoy et al. proposed a decentralized scheduling algorithm that makes nodes autonomously compute their own schedules without signaling overhead. These schedules are allocated to a particular traffic plane and updated automatically as the topology evolves [7]. Vallati et al. assessed the network formation dynamics of 6TiSCH networks and showed that the minimal configuration might lead to long network formation and suboptimal performance of the routing algorithm which may result in a disconnected network. Therefore, they proposed a dynamic resource management algorithm to be executed during network bootstrap, which allows us to minimize the network formation time and also helps in optimizing routing operations to discover better routes [8].

Gonga et al. found that multichannel communication with channel hopping can reduce link burstiness and packet loss correlation significantly over single-hop links. For multi-hop networks, multichannel communication can outperform adaptive routing in sparse networks [23]. Gunatilaka et al. analyzed the impact of channel selection on network topology, routing, and scheduling. They found that there is an inherent tradeoff between channel diversity and route diversity, because using an excessive number of channels may negatively impact routing and scheduling. Then, they proposed some channel and link selection strategies to improve route diversity and network schedulability [24].

### 2.3. Link Quality Estimation for Multichannel

Hermeto et al. thought that the reception rate of broadcast packets provides means to estimate the link quality of different neighbors, even when the data packets use different transmission opportunities. Therefore, they proposed to use the continuous advertisement packets transmitted by the nodes to infer the link quality, which can identify the best routes to the sink during the bootstrapping phase without adding any extra control packets [25]. Considering that the interference pattern is rather dynamic, unpredictable, and highly localized, Kotsiou et al. proposed a localized blacklisting strategy to decide which channels have to be blacklisted or on the contrary recovered. This strategy uses an exponentially weighted moving average to independently measure the packet delivery ratio for each channel [26]. Gall et al. conducted real-world experiments to measure the link quality for wireless nodes placed inside an EV battery pack, in which the packet delivery ratio was used to describe the link quality [27].

Tavakoli et al. proposed an enhanced version of the TSCH protocol together with a distributed channel sensing technique that dynamically detects good-quality channels to be used for communication [28]. Both centralized and distributed technologies are used for LQE. The centralized technology proactively performs energy detections in the idle part of each time slot at the coordinator of the network to follow dynamic interference, while the distributed one is executed by all the nodes in the network, based on their communication history, to detect interference sources that are hidden from the coordinator. Mavromatis et al. proposed a clustering algorithm for high-density TSCH networks, in which the formation of clusters is adapted to the network density and the node selection metric is based on LQI [29].

Farahmand et al. thought that to enable effective blacklisting, especially in highly varying networks, an accurate real-time prediction of the quality of all available channels is of paramount importance. However, previous studies rely on the past records of the channels as an indication of their quality in near future, which evidently cannot be extended to highly dynamic environments. Thus, they proposed a self-supervised approach for training deep neural networks capable of predicting the future behavior of channels [30]. It is shown that the chosen quality assessment metrics such as the PRR, and RSSI greatly affect the performance of the blacklisting scheme.

Boucetta et al. studied the link quality in the TSCH network by analyzing the RSSI and error rates. The objective is to understand the temporal properties of these parameters which are important to select the appropriate channels for critical applications. On this basis, they used machine learning techniques to classify the link quality [31]. Kotsiou et al. proposed a new blacklisting scheme to form a blacklist by making the employed scheduling algorithm blacklist-aware. They identified the bad channels based on several different channel quality metrics, including the noise floor, RSSI, and a spectrum sensing method [32]. Tavakoli et al. proposed a cross-layer low-latency topology management and TSCH scheduling technique, which employs LQI as the quality metric directly. Experimental results show that it reduces the end-to-end communication latency compared to other approaches while keeping communications reliable [33].

In summary, although the works above use some link quality metrics such as PRR, RSSI, and LQI under a multichannel scenario, they pay no attention to the characteristics of these metrics in different channels. Whether the popular LQE metrics and models are valid under multichannel scenarios is not the focus of these works. Therefore, this paper is complementary to them, as our findings could provide new insights into the design of multichannel protocols.

## 3. Popular Link Quality Metrics and Models

Popular link quality metrics were considered in this paper, including PRR and various physical layer metrics such as SNR, RSSI, and LQI [9]. PRR is the most direct metric of link quality. However, the agility of using PRR for LQE is very poor. To estimate the link quality and determine the node locations before network deployment, the spatial distribution model of PRR with distance could be established based on the lognormal path loss model [10], shown as follows:

(1) $$PRR={\left(1-Q\left(\sqrt{2\cdot 10^{\left(P_{t}+L_{C}-PL(d_{0})-10n\log_{10}\left(\frac{d}{d_{0}}\right)+X_{\sigma }-P_{n}\right)/10}\cdot B_{N}/R}\right)\right)}^{Nbit}$$

where *Q*(·) represents the Q function, *P_t_* is the transmit power (dBm), *L_c_* is the signal strength gain (or loss, if its value is negative) in the transceiver (dB), *n* is the path loss exponent characterizing the attenuation rate of wireless signals, *d* is the distance between transmitter and receiver (m), *d*_0_ is the reference distance (usually 1m), *PL*(*d*_0_) is the free-space path loss (dBm) at *d*_0_, *X_σ_* is a normally distributed random variable with zero mean and a standard deviation of *σ* (dB), *P_n_* is the background noise power (dBm), *B_N_* is the noise bandwidth of the transceiver (kHz), *R* is the communication data rate (kb/s), and *Nbit* is the number of bits in the packet. For the 2.4GHz physical layer of IEEE 802.15.4 standard typically used in traditional LLN, the values of *B_N_* and *R* are 384 kHz and 250 kb/s respectively.

The spatial distribution model of PRR is evaluated in this paper. This model is the basis of another important spatial model of low-power links, namely the model for estimating the bounds of the transitional region. If the former is not applicable under the multichannel scenario, the model for estimating the bounds of the transitional region will also fail inevitably. In the following sections, standard deviation *σ* of *X_σ_* was calculated from the variances of RSSI at different distances, *P_n_* used the measured background noise power, and *Lc* and *n* were obtained by using the least squares fitting method.

Using physical layer metrics could solve the agility problem of PRR, as these metrics could be measured per-packet level and are highly correlated with PRR. In practice, the estimated physical layer metrics need to be mapped to PRR to obtain a more granular characterization of link quality [9]. Therefore, the popular models of physical layer metrics and PRR are also evaluated in this paper, which is formulated as follows. For the 2.4GHz physical layer of IEEE 802.15.4 standard typically used in traditional LLN, a theoretical model of SNR and PRR could be established [11], as shown in Equation (2):

(2) $$PRR={\left(1-Q\left(\sqrt{2\times \frac{B_{N}}{R}\times 10^{\frac{SNR}{10}}}\right)\right)}^{Nbit}$$

which is denoted as TH-SNR Model in the following. In addition, logical regression can be used to establish the fitting model of SNR and PRR [12], as shown in Equation (3):

(3) $$PRR=\frac{1}{1+e^{b_{1}\times SNR+b_{2}}}$$

where *b*_1_ and *b*_2_ can be fitted using the experimental data. It is denoted as LR-SNR Model in the following.

Also using logistic regression, the mapping model of RSSI and PRR could be established [13], as shown in Equation (4):

(4) $$PRR=1-\frac{1}{1+c_{1}\times e^{c_{2}\times RSSI+c_{3}}}$$

where *c*_1_, *c*_2,_ and *c*_3_ can be fitted using the experimental data. It is denoted as LR-RSSI Model in the following. For the 2.4GHz physical layer of IEEE 802.15.4 standard typically used in traditional LLN, a simplified theoretical model of RSSI and PRR could be established [14], as shown in Equation (5):

(5) $$PRR={\left(\frac{1}{2}+\frac{1}{2}\times \sqrt{1-e^{-1.78944\times \left(10^{\frac{RSSI-P_{n}+1}{10}}-1\right)}}\right)}^{Nbit}$$

which is denoted as TH-RSSI Model in the following. In addition, a polynomial mapping model between the normalized RSSI and PRR could be established [17]. As the RSSI is normalized to the possible maximum RSSI value, its value ranges are fixed. Therefore, the expression given in [17] was used directly:

(6) $$PRR=-3943.5R_{nor}^{6}+6506.6R_{nor}^{5}-4279R_{nor}^{4}+1430.9R_{nor}^{3}-256.47R_{nor}^{2}+23.77R_{nor}^{1}+0.022$$

where *R_nor_* is the normalized RSSI obtained using the median filter, and its value ranges from 0 to 0.5. As the normalized RSSI is used, this model is self-adaptive to some extent. It is denoted as PN-RSSI Model in the following.

In [15], the mapping model between LQI and PRR was obtained based on the multi-segment linear model, as shown in Equation (7):

(7) $$PRR=\{\begin{array}{cc} 1, & LQI>f_{7}\\ f_{1}\times LQI+f_{2}, & f_{8}<LQI\leq f_{7}\\ f_{3}\times LQI+f_{4}, & f_{9}<LQI\leq f_{8}\\ f_{5}\times LQI+f_{6}, & \;50\leq LQI\leq f_{9} \end{array}$$

where *f*_1_~*f*_9_ can be fitted using the experimental data. It is denoted as ML-LQI Model in the following. In [16], the mapping model between LQI and PRR was obtained using the cubic model, as shown in Equation (8):

(8) $$PRR=\{\begin{array}{cc} 1, & LQI>e_{5}\\ e_{1}\times LQI^{3}+e_{2}\times LQI^{2}+e_{3}\times LQI+e_{4}, & e_{6}<LQI\leq e_{5}\\ 0, & LQI\leq e_{6} \end{array}$$

where *e*_1_~*e*_6_ can be fitted using the experimental data. It is denoted as CU-LQI Model in the following. In [12], the mapping model between LQI and PRR was obtained based on logistic regression, as shown in Equation (9):

(9) $$PRR=\frac{1}{1+e^{g_{1}\times LQI+g_{2}}}$$

where *g*_1_ and *g*_2_ can be fitted using the experimental data. It is denoted as LR-LQI Model in the following. It is worth pointing out that the models above have been derived and analyzed carefully when they were proposed. Therefore, only the final equations of these models are given here and the parameters of these models will be fitted using the measured data we acquired from the experiments.

## 4. Experimental Setup

### 4.1. Experimental Field

**Figure 1:**
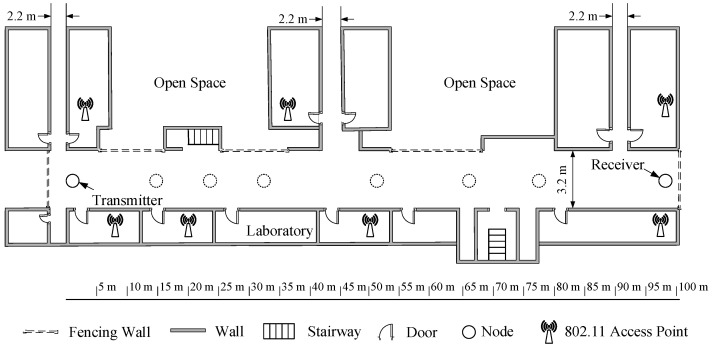
Experimental field: the corridor of the laboratory building with uncontrollable interference sources in nearby rooms.

The corridor of our laboratory building was chosen as the experimental field, as shown in Figure 1. One side of the corridor is the laboratory rooms, and the other side is the walls. The corridor is 106 m long, 3.2 m wide and 2.8 m high. It is relatively clean with few obstacles. Multiple IEEE 802.11 Access Points exist in the laboratory rooms, and their usage pattern is uncontrollable. The purpose of this work is to study the applicability of traditional LQE metrics and models under unknown channel interference. Therefore, controllable interference was not used. TelosB-compatible low-power wireless nodes were used, which employ IEEE 802.15.4 compatible CC2420 radio chips and built-in inverted F PCB antennas [34]. Although TelosB-compatible nodes cannot support true multichannel communication (i.e., use concurrently multiple channels), slow channel hopping schemes could be implemented on top of telosb and other similar platforms, as long as they are compatible with the IEEE 802.15.4 standard. There have been some works about TSCH networks employing telosb and other similar platforms [35,36,37,38,39]. In these works, 16 channels of the IEEE 802.15.4 or a subset of them are used in TSCH mode. Therefore, it is reasonable to use TelosB-compatible nodes to evaluate the applicability of existing LQE metrics and models in multichannel TSCH networks.

### 4.2. Channel Selection

For IEEE 802.15.4, the de facto standard of LLN, IEEE 802.11 is thought to be the most powerful interference source [40]. It will suffer from strong interference if the channel is not allocated properly. Although IEEE 802.15.1 also works in the same band, its interference with IEEE 802.15.4 is much weaker, as the maximum transmit power of IEEE 802.15.1 is much lower than that of IEEE 802.11 [41]. For the experimental setup shown in Figure 1, even though there are many IEEE 802.15.1 devices in nearby rooms, their impacts on the receiver will be covered inevitably by surrounding IEEE 802.11 devices. Therefore, commonly used IEEE 802.11 channels were fully considered when selecting the test channels. Channels 11, 17, 20, 22, and 26 of IEEE 802.15.4 were chosen. Meanwhile, a simple slow channel hopping scheme was designed, and a hopping sequence composed of channels 11, 17, 20, 22, and 26 were used by the transmitter and receiver. The accuracy of link quality models under this simple TSCH scheme was computed and compared with those of single channels.

**Figure 2:**
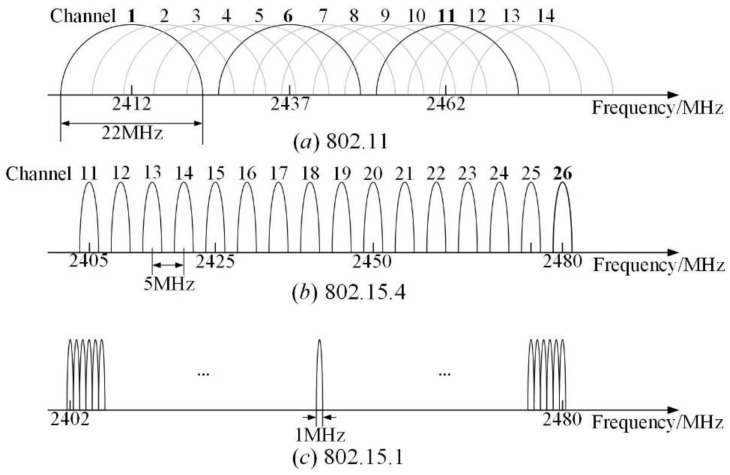
Channels of IEEE 802.11, IEEE 802.15.4, and IEEE 802.15.1.

The five channels selected have significantly different interference levels, as shown in Figure 2. Channel 26 does not overlap with the commonly used IEEE 802.11 channels and is always recognized as interference-free [23]. Channel 20 locates in the middle area of two commonly used IEEE 802.11 channels, which are subject to low interference theoretically. Channels 11, 17, and 22 overlap with the commonly used IEEE 802.11 channels and may suffer from high interference inevitably. The level of interference depends on the usage pattern of corresponding IEEE 802.11 channels. Existing models rarely consider the impact of channels when modeling. Especially, they are basically based on the single interference-free channel 26. Therefore, unless otherwise specified, the measured data under channel 26 will be used uniformly when training the models described in Section 3.

### 4.3. Methodology

One TelosB-compatible node plays as the transmitter, and the other node plays as the receiver. During the experiment, the transmitter was fixed, as shown in Figure 1. Different link qualities were generated by changing the location of the receiver, as shown in Figure 1. The dotted circles represent the different positions of the receiver. A longer distance between the transmitter and receiver produces weaker link quality. As the location of the receiver continuously changed, different interference was also generated, because the interference sources around the receiver also changed. For the simple TSCH scheme designed, time synchronization between the transmitter and receiver was done before the latter was moved to a new location to reduce the complexity.

In each selected channel, the transmitter sent 500 data packets continuously with an interval of 25ms. The default 0dBm transmission power was used. Each data packet carried a sequentially increased serial number. PRR can be obtained by counting the number of packets successfully received. CC2420 provides RSSI and LQI measurements, which can be obtained by accessing corresponding internal registers [42]. In the IEEE 802.15.4 standard, LQI is defined as a quality factor of the received packet. Its value is usually in the range of 50~110 [42]. The higher the LQI, the higher the quality of the data packet. Each time the receiver received a data packet, it relayed the measured RSSI, LQI, and serial number to a PC for analysis. In the following analysis, three main categories of link quality are used, which are good links with PRR ≥ 80%, moderate links with PRR 20% ≤ PRR < 80%, and bad ones with 0 < PRR < 20% [43].

## 5. Spatial Characteristics and Models under Multichannel Scenario

### 5.1. Spatial Characteristics of Low-Power Links

**Figure 3:**
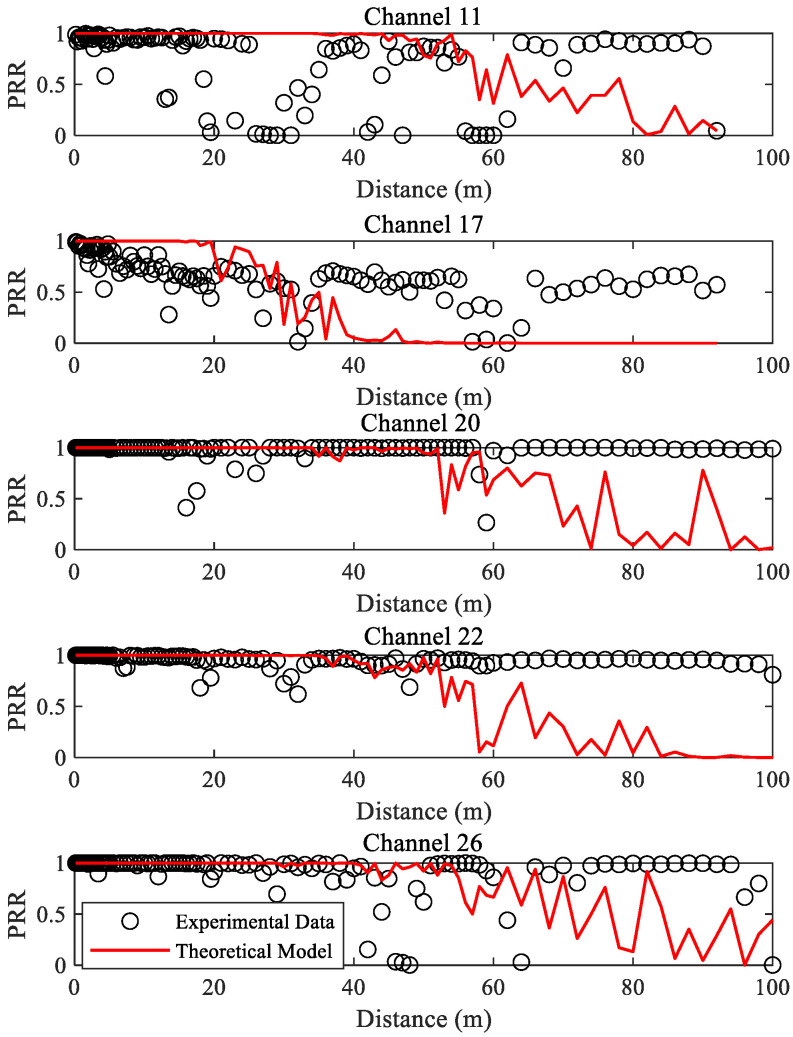
PRR and its spatial distribution model under different channels.

Firstly, spatial characteristics of low-power links under multichannel scenarios are discussed. The changes in PRR with distance in different channels are shown in Figure 3. It can be seen that there is no explicit relation between distance and PRR. On the other hand, the relationships between the distance and PRR are not consistent in different channels. The spatial distributions of PRR vary greatly in different channels. The PRR values corresponding to the same distance are quite different. For example, the PRRs of channel 17 after 10 m are all less than 80%, while only a few PRRs of channel 20 are less than 80%. In addition, the maximum communication distance in different channels also varies. The maximum communication distances of channels 11 and 17 are both 92 m, while PRRs are still very high at 100 m for channels 20 and 22.

In general, the spatial characteristics of low-power links are closely related to the interference level of channels. For channels 11, 17, and 22 are vulnerable to interference, there is almost no case after 10 m where the PRR is 100%. Obviously, the performance of channel 17 is the worst, followed by channel 11, and the performance of channel 22 is close to that of low-interference channels. This also indicates that the interference level of IEEE 802.15.4 channels depends on the usage pattern of corresponding IEEE 802.11 channels [34]. Channel 26, generally recognized as interference-free, does not exhibit the optimal link quality, and its stability is even not as good as that of channel 20. This is similar to the result in [44]. Considering that the frequencies of these channels are not very different, their propagation characteristics should be basically the same. Therefore, it is certain that the spatial characteristics of these channels are mainly determined by their interference level of them.

### 5.2. Spatial Distribution Model of PRR

Next, the impact of channel changes on the spatial distribution model of PRR is analyzed. The main parameters required for the model are shown in Table 1. *P_n_* corresponds to the mean noise in different channels, and other parameters are fitted using the measured data under channel 26. It is obvious that the mean noise is quite different due to the different interference levels of these channels. The mean noise of channels 11, 17, and 22 is higher than that of channels 20 and 26, especially channel 17, in which the mean noise is nearly 6 dBm higher.

With the parameters in Table 1, the changes of PRR with distance under different channels can be obtained, as shown in Figure 3. It can be seen that, as the measured mean noise under different channels is used, there are obvious differences in the spatial distribution model of PRR. It is clear that the estimated PRRs are quite different from the measured ones even under channel 26 of which the measured data was used to fit the model parameters. However, the spatial distribution model can still roughly reflect the speed at which channels with different interference levels enter the transitional region. Channel 17 has the shortest distance to enter the transitional region, followed by channels 11 and 22, and channels 20 and 26 have the longest distance to enter the transitional region. This is because the mean noise will affect the PRR estimated by the spatial distribution model. The higher the mean noise, the faster the PRR will decrease from 1 to 0.

To describe the impact of channel changes on the spatial distribution model quantitatively, Table 2 shows the root mean square errors (RMSEs) of the estimated and measured PRRs under different channels. It can be seen that estimation errors of the spatial distribution model of PRR with distance are very high. The RMSE under channel 11 is even as high as 0.4558, which means that the estimated PRR is completely useless. This also indicates that the spatial distribution model of PRR with distance is not a good approach for LQE. Although introducing the mean noise of channels can roughly reflect the speed of entering the transitional region, it is not enough to accurately characterize the spatial distribution of PRR without accurately modeling the statistical characteristics of channel interference.

## 6. Physical Layer Metrics and Models under Multichannel Scenario

### 6.1. RSSI and RSSI-Based Models

**Figure 4:**
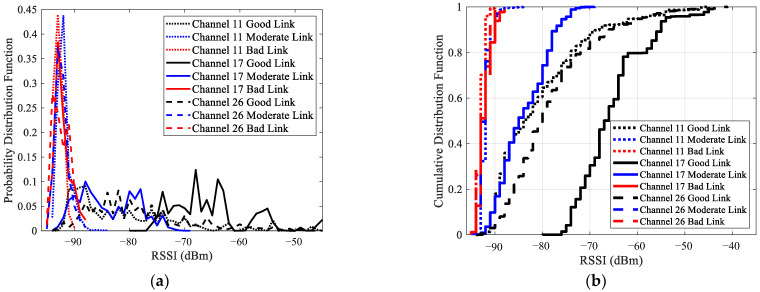
The distributions of RSSI for good links, moderate links, and bad links under the interference-free channel 26, the low interference channel 11, and the high interference channel 17: (**a**) PDF; (**b**) CDF.

The distributions of RSSI for good links, moderate links, and bad links under different channels were analyzed, as shown in Figure 4. Considering the space limitation, only the results of three typical channels are presented. This will not affect the conclusions, as the distributions of RSSI under channels with similar interference are similar. These channels include the interference-free channel 26, the low interference channel 11, and the high interference channel 17. Figure 4a shows the probability density functions (PDFs) of RSSI, while Figure 4b shows the cumulative distribution functions (CDFs) of RSSI. It can be seen that channel changes have little impact on the distribution of RSSI of bad links. However, there are some impacts on the distribution of RSSI of moderate and good links. Channel interference makes the distribution of RSSI shift to the right. The degree of right shift is related to the interference level. The stronger the interference, the greater the right shift. This means that a larger RSSI is required to reach a specific link quality. In other words, the RSSI thresholds established in traditional LLN [9] are no longer valid under the multichannel scenario.

**Figure 5:**
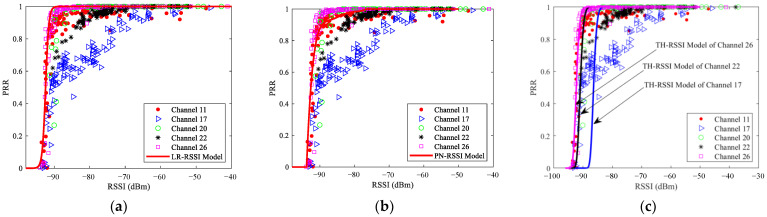
Measured and Estimated RSSI and PRR under different channels: (**a**) The LR-RSSI model; (**b**) The PN-RSSI model; (**c**) The TH-RSSI model.

The measured RSSI and PRR under different channels are shown in Figure 5. It can be seen that the change of PRR with RSSI under different channels is not consistent. On the whole, PRR still increases with the increase of RSSI. When PRR is small (less than 50%), the change of PRR with RSSI in different channels is similar. However, when PRR is large (more than 50%), the change of PRR with RSSI in different channels is obviously different. For channels 20 and 26, PRR can rapidly grow to 100% within the range of −92 dBm < RSSI ≤ −87 dBm. For channels 11 and 22 with low interference, PRR increases to nearly 100% in the range of −92 dBm < RSSI ≤ −72 dBm. For channel 17, which is the most interfered with, PRR slowly increases to nearly 100% in the range of −92 dBm < RSSI ≤ −52 dBm. This also means that the signal power required to achieve 100% PRR is closely related to the interference level. In other words, sufficient reception power is required to resist the adverse effects of interference.

Next, the impact of channel changes on the models of RSSI and PRR is analyzed. For the mean noise required by the theoretical model, the measured data corresponding to each channel was used. For the normalized polynomial model, fitting using the measured data is not needed, as all the required parameters are constant. For the logistic regression model, the fitted parameters are *c*_1_ = 223.2567, *c*_2_ = 2.184, and *c*_3_ = 196.4936. With these parameters, the curves of these models of RSSI and PRR under different channels can be obtained, as shown in Figure 5. Considering that the mean noises of channel 11 and 22 (also channel 20 and 26) are relatively close (as shown in Table 1), which makes the corresponding curves of the theoretical model nearly coincide, only the curves corresponding to channels 17, 22, and 26 are shown in Figure 5c. It is necessary to point out that this does not affect the conclusions. On the other hand, the estimation error of the theoretical model under different channels is still calculated using the corresponding curve of each channel.

It can be seen from Figure 5a that as the logistic regression model is fitted using the measured data of interference-free channel 26, it is only applicable for interference-free channels. For channels with strong interference, such as channels 11, 22, and 17, the logistic regression model will be completely invalid. The same is true for the theoretical model because its modeling process also does not consider the impact of interference. It can be seen from Figure 5c that although the use of mean noise reflects the change of interference caused by channel changes to some extent, it can only make the theoretical model shift left and right as a whole. It cannot fully reflect the impact of channel interference on link quality, especially the degree of the shift to the lower right. Therefore, it is not enough to simply consider the mean noise of the channel. It may be helpful to consider more complex distribution characteristics of interference. It can be seen from Figure 5b that since the normalized polynomial model is trained using the measured data under a specific interference environment [17], it is only consistent under the channels with approximate interference levels, such as channel 11. If the interference level of the channel changes, the model will also be completely invalid.

To describe the impact of channel changes on the models of RSSI and PRR quantitatively, Table 3 shows the RMSEs of estimated and measured PRRs under the single-channel and multichannel scenarios. It is obvious that the accuracy of these models mainly depends on the interference level of the channel. The more seriously the channel interferes, the worse the estimation accuracy. For example, as channel 17 is the most interfered with and the measured data show the most obvious shifts, its error is also the largest. It is obvious that the RMSE under the multichannel scenario is smaller than that of the most interfered channel 17 but larger than that of the interference-free channel 26. It is not difficult to understand. Since these models are typically established under the interference-free channel, channel interference will lead to reduced accuracy of these models. Therefore, if channel hopping is used, the average RMSE will be reduced compared with that under interfered channels, but undoubtedly still larger than that under the interference-free channel in which these models are established.

**Figure 6:**
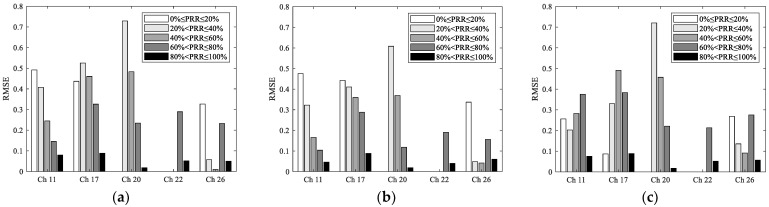
RMSEs of the LR-RSSI model, PN-RSSI model, and TH-RSSI model under different channels: (**a**) RMSE of the LR-RSSI model for different PRR ranges; (**b**) RMSE of the PN-RSSI model for different PRR ranges; (**c**) RMSE of the TH-RSSI model for different PRR ranges.

To describe the estimation error more clearly, errors corresponding to different PRR ranges under different channels are shown in Figure 6. It can be seen that in the range of 40% < PRR ≤ 100%, RMSEs of the most interfered channel 17 are significantly higher than those of other channels. To some extent, this reflects the degree of the shift to the lower right. Interestingly, the RMSE of channel 22 is the smallest. It can be seen from Figure 5 and Figure 6 that this is mainly caused by the smaller number of samples in the transitional region under channel 22. There are almost no samples in the range of 0 ≤ PRR ≤ 60%, which leads to its superiority in calculating the whole estimation error. Similarly, this is also the reason why the RMSE of channel 20 is slightly less than that of channel 26.

In summary, whether the models of RSSI and PRR fitted under a specific channel can be used for other channels mainly depends on whether the interference characteristics of these channels are similar. Since the impact of interference is not considered in modeling, the theoretical model can only be used for channels without interference or with slight interference. Simply considering the mean noise of the channel is not enough to accurately describe the impact of channel interference. It may be helpful to consider more complex distribution characteristics of interference. The normalized polynomial model and the logistic regression model can only be used for channels with the same or similar interference characteristics under which they are modeled. In brief, using the models of RSSI and PRR has to consider the impact of channel changes, otherwise, large estimation errors will occur.

### 6.2. SNR and SNR-Based Models

**Figure 7:**
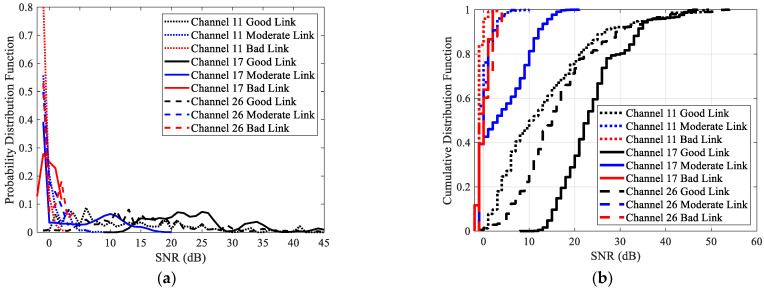
The distributions of SNR for good links, moderate links, and bad links under the interference-free channel 26, the low interference channel 11, and the high interference channel 17: (**a**) PDF; (**b**) CDF.

The distributions of SNR for good links, moderate links, and bad links under different channels were analyzed, as shown in Figure 7. Similarly, only the results of channels 26, 11, and 17 are presented. This will not affect the conclusions, as the distributions of SNR under channels with similar interference are similar. Figure 7a shows the PDFs of SNR, while Figure 7b shows the CDFs of SNR.

It can be seen that channel changes have little impact on the distribution of SNR of bad links. However, there are some impacts on the distribution of SNR of moderate and good links. Like RSSI, channel interference makes the distribution of SNR shift to the right. The degree of right shift is related to the interference level. The stronger the interference, the greater the right shift. This means that larger SNR is required to reach a specific link quality. In other words, the SNR thresholds established in traditional LLN [9] are no longer valid under the multichannel scenario.

**Figure 8:**
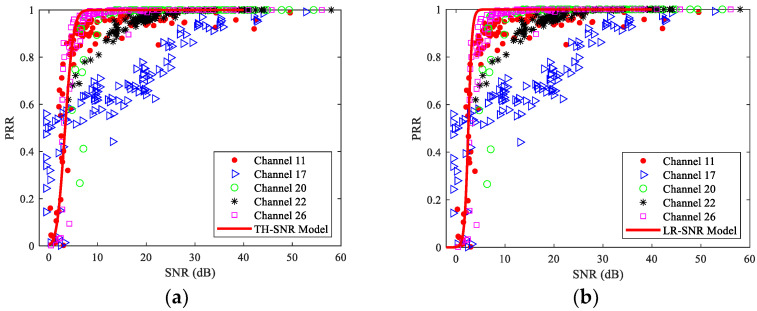
Measured and Estimated SNR and PRR under different channels: (**a**) The TH-SNR model; (**b**) The LR-SNR model.

The measured SNR and PRR under different channels are shown in Figure 8. It can be seen that the change of PRR with SNR under different channels is not consistent. When PRR changes from 50% to 100%, the measured data of channels 11, 17, and 22 shift to the lower right with varying degrees. In other words, compared with the interference-free channel, the SNR required to achieve 100% PRR is much larger for these channels. Meanwhile, it is rather difficult to achieve 100% PRR for channel 17. In contrast, PRR can rapidly grow to 100% under channels 20 and 26. However, compared with channel 26, the measured data under channel 20 shifts to the right as a whole. On the other hand, when PRR changes from 0 to 50%, the measured data under channel 17 obviously shift to the left. This is significantly different from Section 6.1, for which the change of PRR with RSSI in different channels is similar. This is mainly caused by the strongest interference under channel 17. As can be seen from Figure 4, the RSSIs with small PRRs (bad links) are less affected under interfered channels. However, the measured noise power increase under these channels, which makes the computed SNR shift to the left.

Next, the impact of channel changes on the theoretical and logistic regression model of SNR and PRR is analyzed. As all the parameters required for the theoretical model are constant, fitting using the measured data is not needed. For the logistic regression model, the fitted parameters are *b*_1_ = -2.5773 and *b*_2_ = 6.4676. With these parameters, the theoretical and logistic regression curves of PRR with SNR under different channels can be obtained, as shown in Figure 8. It can be seen that these models are only applicable to channel 26. Although the pattern of the measured data under channel 20 is basically the same as these models, it shows an obvious right shift as a whole. On the other hand, the measured data under channels 11, 17, and 22 significantly deviate from the estimated ones of these two models, especially under channel 17 which is the most interfered.

To describe the impact of channel changes on these models quantitatively, Table 4 shows the RMSEs of estimated and measured PRRs under the single-channel and multichannel scenarios. The RMSE under the multichannel scenario is smaller than that of the most interfered channel 17 but larger than that of the interference-free channel 26. It is not difficult to understand as these models are also established under the interference-free channel. Therefore, if channel hopping is used, the average RMSE will be reduced compared with that under interfered channels, but undoubtedly still larger than that under the interference-free channel in which these models are established.

**Figure 9:**
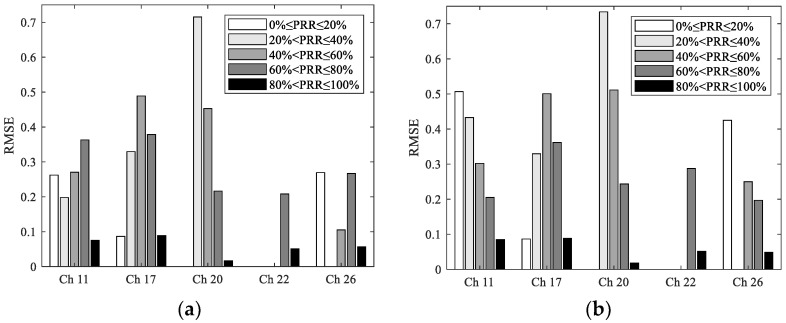
RMSEs of the TH-SNR model and LR-SNR model under different channels: (**a**) RMSE of the TH-SNR model for different PRR ranges; (**b**) RMSE of the LR-SNR model for different PRR ranges.

To describe the estimation error more clearly, errors corresponding to different PRR ranges under different channels are shown in Figure 9. It is obvious that the accuracy of these models mainly depends on the interference level of the channel. For example, as channel 17 is the most interfered with and the measured data show the most obvious shifts, its error is also the largest. Similarly, the fact that the RMSE of channel 22 is the smallest and the RMSE of channel 20 is slightly less than that of channel 26 is also mainly caused by the small number of samples in the transitional region for these two channels, as shown in Figure 8 and Figure 9.

In summary, whether the models of SNR and PRR fitted under a specific channel can be used for other channels mainly depends on whether the interference characteristics of these channels are similar. Since the impact of interference is not considered in modeling, the theoretical model can only be used for channels without interference or with slight interference, such as channels 26 and 20. The logistic regression model can only be used for channels with the same or similar interference characteristics under which it is modeled. In brief, using the models of SNR and PRR has to consider the impact of channel changes, otherwise, large estimation errors will occur.

### 6.3. LQI and LQI-Based Models

**Figure 10:**
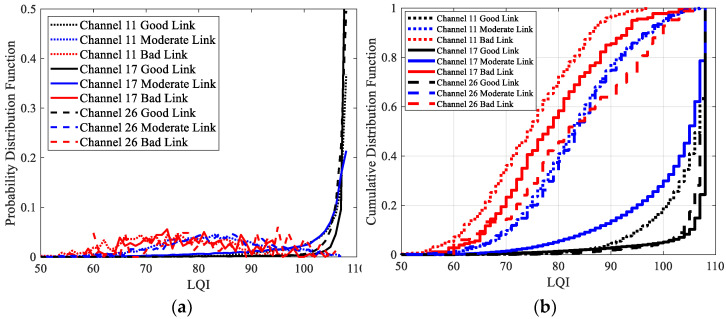
The distributions of LQI for good links, moderate links, and bad links under the interference-free channel 26, the low interference channel 11, and the high interference channel 17: (**a**) PDF; (**b**) CDF.

The distributions of LQI for good links, moderate links, and bad links under different channels were analyzed respectively, as shown in Figure 10. Similarly, only the results of channels 26, 11, and 17 are presented. This will not affect the conclusions, as the distributions of LQI under channels with similar interference are similar. Figure 10(a) shows the PDFs of LQI, while Figure 10(b) shows the CDFs of LQI. It is clear that the distributions of LQI of all the good links, moderate links, and bad links are affected by channel change. Among them, the impacts on moderate links are the greatest. Due to the obvious upper bound of LQI, the impacts of channel change on good links are not as obvious as RSSI and SNR.

Like RSSI and SNR, channel interference makes the distribution of LQI shift to the right. The degree of right shift is related to the interference level. The stronger the interference, the greater the right shift. This means that a larger LQI is required to reach a specific link quality. In other words, the LQI thresholds established in traditional LLN [9] are no longer valid under the multichannel scenario. More seriously, as can be seen from Figure 10, the LQIs of moderate links overlap with those of good links substantially under the most interfered channel 17. It means a sufficiently large LQI can no longer guarantee a good link in channels with strong interference. In other words, the LQE capability of LQI has even been completely lost in channels with strong interference.

**Figure 11:**
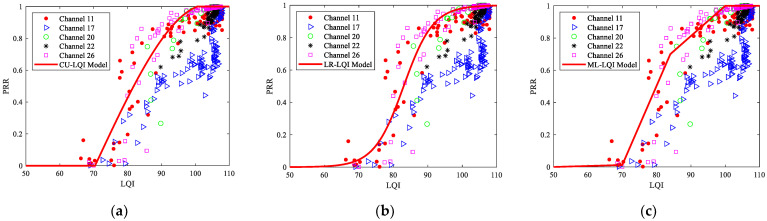
Measured and Estimated LQI and PRR under different channels: (**a**) The CU-LQI model; (**b**) The LR-LQI model; (**c**) The ML-LQI model.

The measured LQI and PRR under different channels are shown in Figure 11. It can be seen that the change of PRR with LQI under different channels is not consistent. On the whole, PRR still increases with the increase of LQI. When PRR is small (less than 50%), the change of PRR with LQI in different channels is almost similar. However, when PRR is large (more than 50%), the change of PRR with LQI in different channels is obviously different. For interference-free channels or channels with low interference, PRR gradually increases to 100% in the range of 80 ≤ LQI ≤ 110. For channels with strong interference, PRR corresponding to the range of 80 ≤ LQI ≤ 110 is significantly lower than that of interference-free channels or channels with low interference. Meanwhile, PRR will jump to 100% within the range of 104 ≤ LQI ≤ 110. This means that LQI, which is thought to have higher resolution in traditional LLN, is inferior to SNR and RSSI under interfered channels.

Next, the impact of channel changes on the models of LQI and PRR is analyzed. All the multi-segment linear model, cubic model, and logistic regression model of LQI and PRR were trained with the measured data under channel 26. The fitted parameters are shown in Table 5. With these parameters, the curves of these models of LQI and PRR under different channels can be obtained, as shown in Figure 11. It can be seen that the curves of these models are basically consistent with the measured data under channel 26. However, there are large differences between the estimated PRRs of these models and the measured PRRs under other channels, especially for channels with strong interference. When the interference level changes, these three models will be completely invalid.

To describe the impact of channel changes on the models of LQI and PRR quantitatively, Table 6 shows the RMSEs of estimated and measured PRRs under different channels. It is obvious that the accuracy of these models also mainly depends on the interference level of the channel. The more seriously the channel is interfered with, the worse the estimation accuracy. Meanwhile, the RMSE under the multichannel scenario is smaller than that of the most interfered channel 17 but larger than that of the interference-free channel 26. The reason is the same as described for RSSI and SNR, so it is omitted here.

**Figure 12:**
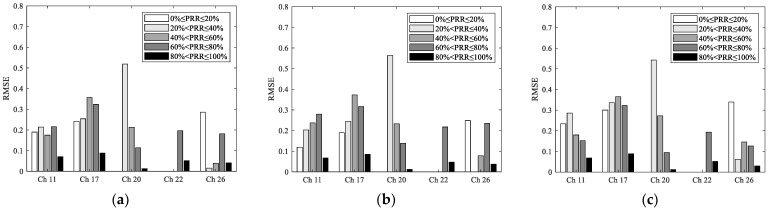
RMSEs of the CU-LQI model, LR-LQI model, and ML-LQI model under different channels: (**a**) RMSE of the CU-LQI model for different PRR ranges; (**b**) RMSE of the LR-LQI model for different PRR ranges; (**c**) RMSE of the ML-LQI model for different PRR ranges.

To describe the estimation error more clearly, errors corresponding to different PRR ranges under different channels are shown in Figure 12. It can be seen that in the range of 40%<PRR≤100%, RMSEs of the most interfered channel 17 are significantly higher than those of other channels, which is similar to the conclusions for SNR and RSSI. In addition, the reason why the RMSEs of channel 20 and 22 is less than that of channel 26 is still that fewer samples exist in the transitional region for these two channels.

In summary, the LQE capability of LQI will be affected by the interference level of the channel considerably. It is inferior to SNR and RSSI under interfered channels, although it is thought to have the highest resolution in traditional LLN. Meanwhile, whether the models of LQI and PRR fitted under a specific channel can be used for other channels mainly depends on whether the interference characteristics of these channels are similar. These models can only be used for channels with the same or similar interference characteristics under which they are modeled. As they do not consider the impact of channel changes, they will be completely invalid under channels with strong interference.

## 7. Generality of The Conclusions

**Figure 13:**
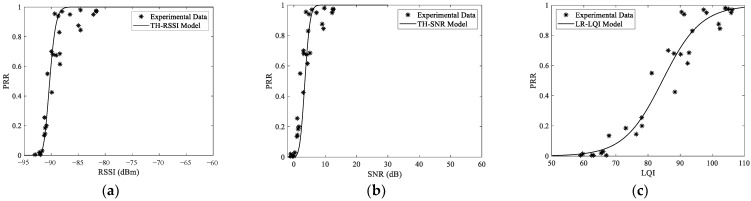
Typical link quality metrics and models with the 5.8 GHz UAV: (**a**) RSSI and its theoretical model with PRR, (**b**) SNR and its theoretical model with PRR, (**c**) LQI and its logistic regression model with PRR.

**Figure 14:**
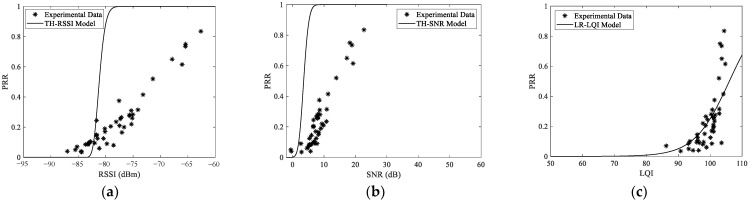
Typical link quality metrics and models with the 2.4 GHz UAV: (**a**) RSSI and its theoretical model with PRR, (**b**) SNR and its theoretical model with PRR, (**c**) LQI and its logistic regression model with PRR.

To verify the generality of the conclusions above, a completely different setup was designed. In this setup, an open playground with little WiFi interference was chosen. To emulate different interference scenarios, two unmanned aerial vehicles (UAV) operating in the 2.4 GHz and 5.8 GHz ISM bands respectively were used. Undoubtedly, the 5.8 GHz UAV should not interfere with the communication of TelosB nodes. However, as the 2.4 GHz UAV uses frequency hopping technology for communication, it will inevitably interfere with all the channels of TelosB nodes. The same methodology as Section 4.3 was employed. In the process of measurement, the UAVs were always placed near the receiver. The measured data and corresponding models with the 5.8 GHz UAV are shown in Figure 13, while those with the 2.4 GHz UAV are shown in Figure 14. For clarity, only one model was plotted and compared with the measured data of channel 26 for each metric. This will not affect the conclusions.

Obviously, the main conclusions above are still valid. The link quality estimation capability of typical metrics will be affected by the interference level of the channel, which makes the conclusions obtained under the single-channel scenario no longer valid. The thresholds of popular link quality metrics established in traditional LLN will lead to incorrect judgment of link quality under the multichannel scenario. Meanwhile, LQI is inferior to SNR and RSSI under interfered channels, although it is thought to have the highest resolution in traditional LLN. In particular, its LQE capability has even been completely lost in channels with strong interference. On the other hand, traditional LQE models are basically not adaptive to radio interference and channel change. They are only valid for channels with similar interference under which they are modeled. If traditional link quality models are directly used under a multichannel scenario, the link quality will be overestimated inevitably.

## 8. Conclusions

Due to its resilience to wireless interference and multi-path fading, multichannel communication can effectively improve the reliability of LLN under cross-technology interference, which has received extensive attention from academia and industry. However, existing LQE metrics and models are generally established and verified under the single-channel scenario [10,11,12,13,14,15,16,17,18,19,20], and most of them are under the interference-free channel [10,11,12,13,14,15,16,18,19]. In the multichannel scenario, as the channels used will change from time to time, whether these metrics and models are still applicable is hard to judge directly because of the lack of corresponding studies.

In view of this, this paper analyzes the applicability of popular LQE metrics and models in many different channels empirically. Results show that the interference level of the channel will affect the capability of traditional LQE metrics. In particular, the LQE capability of LQI will be completely lost in strong interference channels, although it is thought to have higher resolution than SNR and RSSI in traditional LLN. Traditional LQE models including theoretical and empirical models are basically not adaptive to radio interference and channel change. They are only effective for channels with similar interference levels. Although some models try to provide adaptability by introducing the environmental noise or using normalized physical layer metrics, they are unable to achieve the expected effect because this cannot accurately model the characteristics of interference.

Therefore, traditional LQE metrics and models cannot be directly used in the multichannel scenario. On the one hand, the thresholds of popular link quality metrics established in traditional LLN will lead to incorrect judgment of link quality under a multichannel scenario. On the other hand, the link quality will be overestimated inevitably if traditional link quality models are directly used under a multichannel scenario. In future works, it is necessary to deeply analyze the statistical characteristics of existing LQE metrics in multiple typical channels and design channel and interference adaptive LQE metrics and models to support effective multichannel communication in LLN.

## Figures and Tables

**Table 1:** Main parameters required for the spatial distribution model of PRR.

**Parameter**	***L_c_***	***n***	***σ***	***P_n_* (Ch 11)**
**Value**	36.6957	1.5896	1.4725	−95.76 dBm
**Parameter**	***P_n_* (Ch 17)**	***P_n_* (Ch 20)**	***P_n_* (Ch 22)**	***P_n_* (Ch 26)**
**Value**	−90.35 dBm	−96.27 dBm	−94.89 dBm	−96.23 dBm

**Table 2:** RMSEs of the spatial distribution model of PRR under different channels.

**Channel**	**11**	**17**	**20**	**22**	**26**
**RMSE**	0.4558	0.3819	0.3263	0.3677	0.3130

**Table 3:** RMSEs of the models of RSSI and PRR under the single channel and multichannel scenarios.

Channel	11	17	20	22	26	Multichannel
**LR-RSSI**	0.2324	0.3435	0.1020	0.0800	0.1049	0.1985
**PN-RSSI**	0.2117	0.2920	0.0793	0.0581	0.1057	0.1723
**TH-RSSI**	0.1622	0.3512	0.0984	0.0697	0.1038	0.1856

**Table 4:** RMSEs of the models of SNR and PRR under single-channel and multichannel scenarios.

Channel	11	17	20	22	26	Multichannel
**TH-SNR**	0.1615	0.3482	0.0975	0.0689	0.1026	0.1840
**LR-SNR**	0.1662	0.3636	0.0969	0.0687	0.1324	0.2042

**Table 5:** Parameters of the models of LQI and PRR.

**Parameter**	***e*_1_**	***e*_2_**	***e*_3_**	***e*_4_**	***e*_5_**	***e*_6_**
**Value**	−0.000013918	0.002948	−0.1626	1.6928	70.4	100.5
**Parameter**	***f*_1_**	***f*_2_**	***f*_3_**	***f*_4_**	***f*_5_**	***f*_6_**
**Value**	0.01875	−0.875	0.04913	−3.4269	0.00061	−0.0305
**Parameter**	***f*_7_**	***f*_8_**	***f*_9_**	***g*_1_**	***g*_2_**	
**Value**	100	84	70	−0.2331	19.3453	

**Table 6:** RMSEs of the models of RSSI and PRR under single-channel and multichannel scenarios.

Channel	11	17	20	22	26	Multichannel
**CU-LQI**	0.1200	0.2859	0.0601	0.0671	0.0889	0.1486
**LR-LQI**	0.1131	0.2834	0.0658	0.0673	0.0865	0.1468
**ML-LQI**	0.1319	0.2951	0.0650	0.0666	0.0932	0.1548

## Data Availability

Not applicable.
